# Zebrafish sleep displays distinct sub-states

**DOI:** 10.1016/j.isci.2026.115004

**Published:** 2026-02-12

**Authors:** Richa Tripathi, Grigorios Oikonomou, Olivia Eliopoulos, David A. Prober, Geoffrey J. Goodhill

**Affiliations:** 1 Departments of Developmental Biology and Neuroscience, Washington University in St. Louis, St. Louis, MO 63110, USA; 2 Tianqiao and Chrissy Chen Institute for Neuroscience, Division of Biology and Biological Engineering, California Institute of Technology, Pasadena, CA 91125, USA

**Keywords:** Behavioral neuroscience, Neuroscience

## Abstract

Sleep is an essential and evolutionarily conserved behavior. While mammals and several other species have been shown to exhibit well-defined sleep sub-states, it remains unclear to what extent such differentiation exists across the animal kingdom. Here we show, using long-term behavioral data combined with Hidden Markov Modeling, that larval zebrafish display two behaviorally defined sleep-related sub-states: a low-activity state consistent with light sleep and a quiescent state consistent with deep sleep. These are likely analogous to light and deep NREM sleep in mammals. Although both states occur primarily at night, arousal responses differ by sleep sub-state, and sleep deprivation induces deep sleep-dominated rebound. Moreover, the proportions of deep and light sleep are selectively altered by genetic and pharmacological manipulations of melatonin, serotonin, and norepinephrine signaling, offering new insights into how these neuromodulators shape sleep architecture. These results support zebrafish as a tractable model for dissecting the regulation and function of sleep sub-states. More broadly, they demonstrate that structured, multi-state sleep is a conserved feature of vertebrate behavior.

## Highlights

• Hidden Markov models identify deep and light sleep states in freely behaving zebrafish
• Light exposure is required to maintain high-activity waking behavior
• Melatonin mediates the circadian oscillation of deep sleep
• Serotonin and noradrenaline differentially regulate sleep depth

## Graphical abstract

**Figure:**
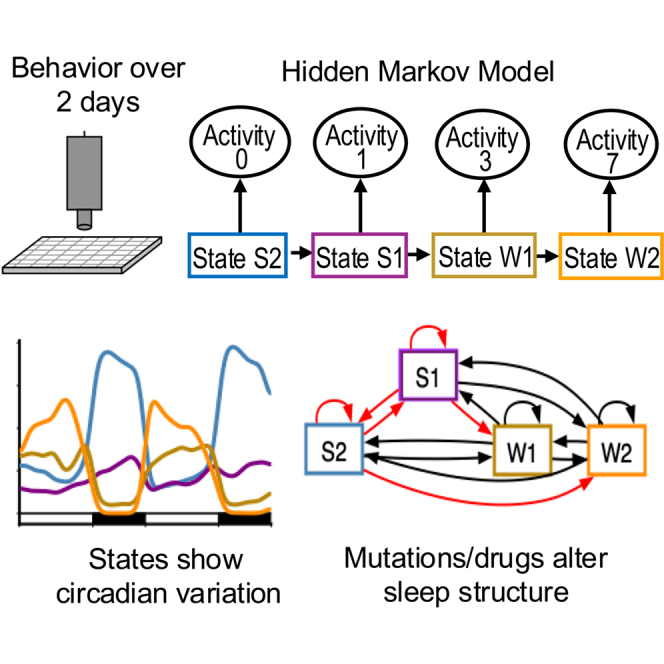

## Introduction

Sleep is regulated by a complex interplay of homeostatic and circadian mechanisms.^1^ Based on the behavioral definition of sleep as a rapidly reversible and homeostatically regulated state of behavioral quiescence that is associated with an increased arousal threshold, sleep has been observed in all studied animals. However, despite being an evolutionarily conserved behavior,^2^^,^^3^ the mechanisms that underlie sleep remain unclear.^4^^,^^5^ Mammalian sleep is not a uniform state but consists of distinct sub-states, which are linked to unique physiological processes spanning cellular, circuit, and systems levels.^6^^,^^7^^,^^8^^,^^9^ Sleep sub-states have also been identified in birds,^10^ reptiles,^11^^,^^12^^,^^13^ cephalopods,^14^ jumping spiders,^15^ and flies,^16^^,^^17^^,^^18^ however whether they also exist in other species remains unclear.^2^^,^^5^^,^^19^

Zebrafish are diurnal vertebrates that offer key advantages for studying sleep, including at the larval stage, their compatibility with whole-brain neuronal imaging and large-scale behavioral assays, and ease of genetic and pharmacological manipulation.^20^^,^^21^^,^^22^ However, the use of non-invasive electrophysiology, such as EEG, the gold standard for identifying sleep sub-states in humans, is challenging in larval zebrafish. Recent work based on neural calcium imaging has suggested the presence of sleep sub-states.^23^ However, the use of highly constrained environments and visible wavelengths of light for fluorescence excitation leaves open the possibility of confounds, including stress responses, suppression of sleep by visible light, and homeostatic rebound effects.

An alternative approach for identifying sleep sub-states is based just on behavior, in particular, overall levels of motor activity. Sleep is a quiescent state that should not be disturbed during measurement, thus to preserve its natural dynamics, sleep is ideally studied non-invasively in freely behaving animals.^24^^,^^25^^,^^26^ This approach not only allows for the characterization of normal sleep and its sub-states under standard conditions, but also leverages the advantages of zebrafish for the high-throughput investigation of how genetic and pharmacological perturbations affect sleep.^27^^,^^28^

Here, we exploit large-scale, long-term behavioral data of larval zebrafish combined with Hidden Markov Models (HMMs) to reveal distinct sleep sub-states. Using the Bayesian Information Criterion (BIC), we show that larval zebrafish locomotor activity is robustly optimally classified into 4 states, of which two are sleep sub-states (occurring primarily at night), and two are wake sub-states (occurring primarily during the day). Arousal assays and sleep deprivation experiments demonstrate that the two sleep sub-states match the characteristics of deep and light sleep states identified in other species. We then use this approach to reveal how genetic, pharmacological, and environmental manipulations differentially affect sleep sub-states. Melatonin-deficient fish showed reduced deep sleep with compensatory increases in light sleep under standard day/night conditions, and the loss of circadian regulation of sleep that occurs in melatonin-deficient fish in free-running constant dark conditions was due to the loss of circadian oscillation of the deep sleep state. Strikingly, constant darkness eliminated the highest-activity wake state during subjective day, demonstrating that light-induced arousal is required for this state. Manipulations of serotonin and noradrenaline signaling similarly shifted the balance between deep and light sleep, with serotonin promoting deep sleep and noradrenaline suppressing it.

Together, this work introduces a robust framework for identifying and analyzing sleep sub-states in larval zebrafish and provides new insights into how neuromodulators shape sleep architecture in a diurnal vertebrate.

## Results

**Figure 1:**
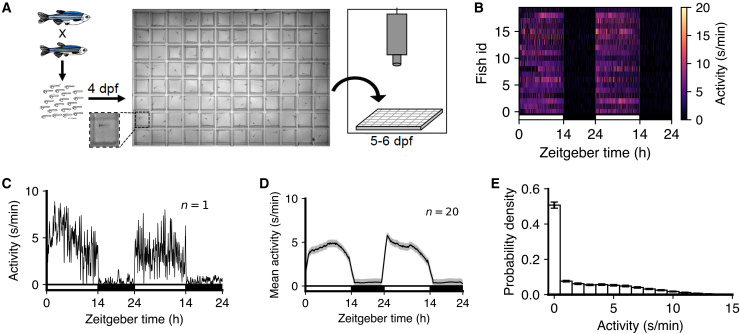
Monitoring larval zebrafish activity over 48-h day-night cycles (A) Overview of experimental assay. (B) Heatmap of locomotor activity starting at 5 dpf (n = 20 fish). (C) Locomotor activity for one representative fish. (D) Mean activity for all fish, with SEM shown in gray. (E) Distribution of locomotor activity for all fish (mean ± sem). In (B–D) the white and black horizontal bars depict the lights-on and lights-off periods on both days, respectively.

Unless stated otherwise, all analyses were based on the following experimental paradigm.^25^ Larval zebrafish, raised under 14:10-h light-dark (LD) conditions, were placed into 96-well plates at 4 days post-fertilization (dpf), and allowed to acclimate to their new environment overnight. Locomotor activity was then monitored for two days and nights, using infrared light and an infrared camera at 30 Hz, starting when white lights turned on at 9 a.m. at 5 dpf (Figure 1A). Locomotor activity was based on pixel changes between consecutive frames (see STAR Methods) and was quantified as seconds of locomotor activity per minute. We first analyzed wild-type (WT) fish, which show higher levels of locomotor activity during the day, when white lights are on, than at night, when white lights are off (Figures 1B–1D). Mean locomotor activity had a heavy-tailed distribution with a peak at zero (Figure 1E).

### Larval zebrafish display 2 sleep sub-states and 2 wake sub-states

To model these data, we used HMMs. An HMM assumes that a system is progressing over time through a sequence of internal states which are not directly observed (“hidden”). Each state is associated with a distribution (here assumed to be Poisson) over observation probabilities, where here observations are activity in s/min. At each 1-min time step, the system can stay in the same state or move to any of the other states with particular probabilities. The Markov assumption, which makes model fitting tractable, is that these transition probabilities, and thus the probability of the next state, depend only on the current state, and not on any longer history of the system. Using well-established algorithms, it is straightforward to fit the sequence of states and transition probabilities that is most likely to have generated a particular sequence of observations. While the number of underlying states is assumed when fitting the model, an optimal number of states can be determined using the Bayesian Information Criterion (BIC). This trades off the better data fit that inevitably arises from models with more parameters against the increased complexity of the model, in order to find the simplest model that fits the data well.

However, HMM fitting is not a deterministic process and may get stuck in local minima. To help ameliorate this issue, in all cases reported here we ran the fitting algorithm 1000 times on each fish, and selected the model with the maximum log likelihood of the fitted data (Figure S1A). To determine if the amount of data available for each fish (48 h × 1 min = 2880 observations) was sufficient to reliably recover model parameters, we first fitted 4-state HMMs to fish data. From these fitted parameters, we then generated surrogate observation sequences of lengths ranging from 10 h to 72 h, and fitted HMMs to these. By 48 h observations, the difference between the fitted and ground-truth parameters was small (Figure S1B), giving us confidence that the length of our data was sufficient for reliable model fitting.

**Figure 2:**
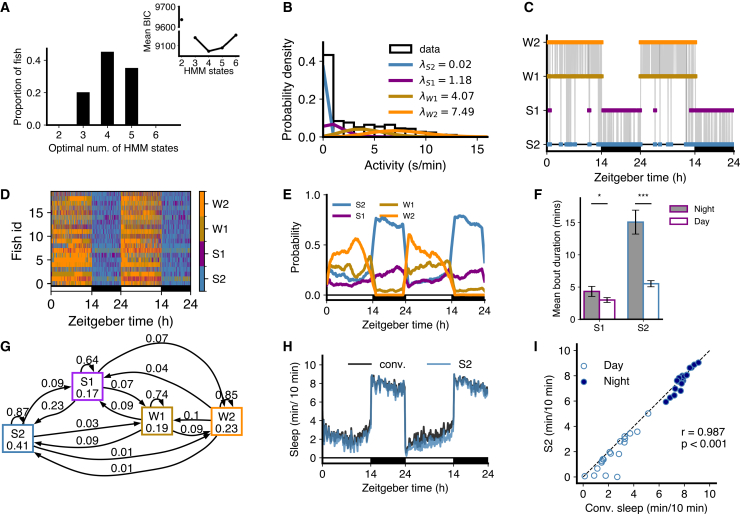
An HMM with 4 states best fits the data for WT fish (A) The distribution of optimal number of states. Inset: Mean BIC as a function of number of HMM states. (B) The four means (λs; units s/min) characterize the Poisson fits to fish locomotor activity for each state. (C) The most likely state sequence inferred from the data for a single example fish, based on parameters of a four-state HMM fit. (D) State sequences for 20 fish over 48 h at 5–6 dpf. (E) Variation of mean posterior probabilities of each state as a function of time, shown as a moving window average. (F) Both mean S1 and mean S2 bout durations were longer at night than during the day (Wilcoxon signed-rank test, ∗p<0.05, ∗∗∗p<0.001, with Benjamini-Hochberg multiple comparisons correction). (G) The transition probabilities averaged across all fish's individual 4-state HMM fit. (H) Sleep amounts obtained based on the conventional definition of sleep (1 min of no locomotor activity, black) compared to the most-likely state sequence from 4 HMM fit. (I) Comparison of HMM-defined S2 sleep with conventionally defined sleep during day and night. Filled and empty circles represent night and day sleep amounts, respectively (one dot per fish). In (C, D, E, and H), the white and black horizontal bars depict the lights on and lights off periods, respectively. r and *p*-value are for Pearson’s correlation for the fit to the line y=x.

To determine the optimal number of states, we fit HMMs containing 2–6 states to each fish and used BIC to determine which number of states was optimal. Interestingly, there was some variation in the optimal number between fish (Figure 2A). To determine if any of this variability was due to model fitting getting stuck in local minima, we generated 100 state transition sequences and associated observations from a 4 HMM fit to a fish, refit those observations with HMMs with 2–6 states, and again used BIC to determine the optimal number of states. In 94% of cases, the answer was again 4, indicating that the variation in optimal number of states between fish represents genuine biological variability rather than variability in model fitting (Figure S1C). Consistent with this notion, there was considerable variation in sleep/wake behaviors among different animals (Figure 1B). However, despite this variability, the modal optimal number of states across fish was 4, and the mean BIC value averaged over fish also had an optimum at 4 (Figure 2A). For consistent subsequent analysis of HMM parameters, we used 4-state HMM fits for all fish unless otherwise stated.

For these 4 states the fitted means of the Poisson observation distributions (i.e., the mean amount of locomotor activity in each state, which we refer to as λ) were roughly 0, 1, 4, and 8 s/min (Figure 2B). Following the terminology of^17^ we refer to these states as S2, S1, W1, and W2, respectively. S2 and S1 were primarily observed at night, and so we refer to them as sleep states, with wake states W1 and W2 observed primarily during the day (Figures 2C–2E and S2).

The mean length of S1 and S2 states was also longer at night than during the day (Figure 2F). The HMM transition diagram for the average fit over all fish is shown in Figure 2G (discussed in next section). As a robustness check we also refit the data with zero-inflated Poisson distributions, and results were very similar (Figure S3). We also checked whether the HMM results are affected by fitting only the first 24 h, but found no significant differences (Figure S1D).

The complete set of λ values for all fish is shown in Tables S1–S3. Table S1 includes fish for which 4 states were optimal. Table S2 includes fish for which 3 states were optimal, and also shows the λ values for the 4-state fit. In the 3-state fit, these fish generally had only one high-activity (wake) state. Table S3 includes fish for which 5 states were optimal, and also shows the λ values for the 4-state fit. In the 5-state fit, these fish generally had an additional low-activity (sleep) state.

Sleep in larval zebrafish has been defined as any period of immobility longer than 1 min, because this is associated with an increase in arousal threshold.^25^^,^^29^ Comparing the occupancy of the S2 state with this conventional definition produced a good match (Figures 2H and 2I). Note that fitting HMM states is not equivalent to simply defining states via locomotor activity thresholds. Consider, for instance, a 1 min bin with no activity. The relative probabilities that this was produced by Poisson distributions with the means for S2, S1, W1, and W2 found for the WT fish (0.02, 1.18, 4.07, 7.49 s/min, respectively) are 90.1%, 7.9%, 1.9%, and 0.03%, respectively. The model determines the most likely state assignment based on the complete sequence of state assignments, taking into account the tendency to persist in the same state between subsequent bins. Thus, a bin with no activity could be classified as either S1 or S2 (and very occasionally W1). Across this set of fish, the fraction of bins with activity 0 s/min assigned states S2 and S1 were 0.84 and 0.16, respectively, and the fractions of bins with activity 1 s/min assigned as S2 and S1 were 0.09 and 0.82, respectively (with the remainder assigned to W1 or W2). This explains why the points in Figure 2I lie slightly below the y = x line: the activity-0 bins S2 loses to S1 are not fully compensated for by the activity-1 bins S2 gains from S1, and thus the total S2 is slightly less than conventional sleep, i.e., the sum of all activity-0 bins.

The difference between the HMM model and defining states by simple activity thresholds is further emphasized by examining the distributions of activity assigned to each state for an example fish (Figure S4). There is considerable overlap between the distributions for S1 and W1 states, and the state assignment of a particular activity level depends on the broader context in which that activity level occurs. For instance, bins with activity of 2 s/min are assigned almost equally to S1 and W1 states. However, at night they are assigned 84% of the time to S1 and only 15% of the time to W1, but during the day the assignment is 33% to S1 and 48% to W1 (Figure S4G). Thus, the HMM captures contextual structure in the data that cannot be recovered by fixed activity thresholds.

For comparison, we also examined the biological consequences of fitting these data with a 2-state HMM (Figure S5), despite its much worse log likelihood value. Unsurprisingly, the fitted λs were intermediate between S1 and S2 for the “sleep” state and intermediate between W1 and W2 for the “wake” state. Because of the relatively high λ for the sleep state, the amount of time spent in this state was a poor match to conventionally defined sleep. Thus, a simple 2-state model was both statistically suboptimal and biologically less informative compared to a 4-state model.

### S1 is a light sleep state, and S2 is a deep sleep state

Mammalian sleep consists of REM and NREM periods, and NREM sleep can be subdivided into distinct states characterized by different sleep depth. Deep NREM sleep has several behavioral properties that distinguish it from light NREM sleep. First, transitions to wake states are more likely to occur from light than deep sleep.^17^ Second, although normally deep and light sleep states alternate, deep sleep is more prevalent under conditions of high homeostatic sleep pressure, such as during rebound sleep following sleep deprivation.^30^ Third, animals in deep sleep are less responsive to an arousing stimulus than animals in light sleep.^31^ We tested each of these properties for zebrafish to determine whether S2 represents a deep sleep state and S1 a light sleep state.

**Figure 3:**
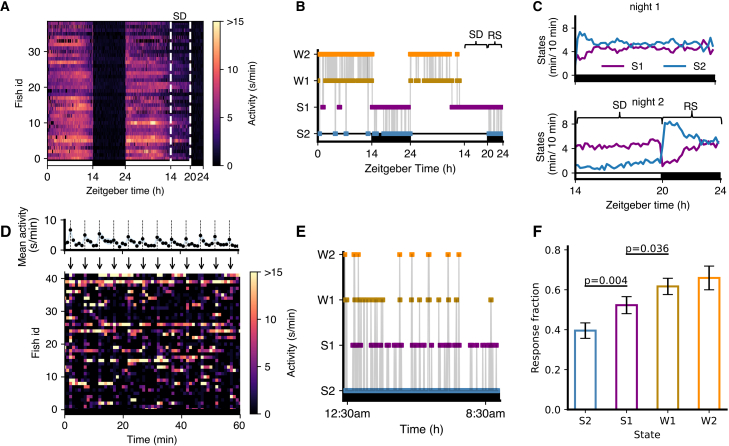
Fish in S1 vs. S2 states show different responses to arousing stimuli and sleep deprivation (A–C) Sleep deprivation experiment. (A) Fish were subjected to sleep deprivation (SD) on night 2, when lights were kept on for the first 6 h at night (shown within dashed white lines), and then turned off for the last 4 h of the night. (B) State sequence for an example fish over 48 h (RS, rebound sleep). (C) Comparison of S2 and S1 during night 1 and night 2. (D–F) Arousal experiment. (D) An example 60 min of data with stimuli every 5 min (arrows) shown as a color map, with mean and standard error of mean locomotor activity over fish shown in the upper plot. (E) A state sequence for an example fish over the night. In (D and E), the black horizontal bars indicate that the lights were off. (F) Fish shows a higher proportion of responses (baseline-corrected) to stimuli when in the S1 state than in the S2 state, and when in the W1 state compared to the S1 state. Data are represented as mean ± SEM. The *p* values shown are from t-tests with Benjamini-Hochberg multiple comparisons correction. When arcsine-transformed response fractions were used, the *p* values were 0.004 and 0.030, respectively.

First, the HMM transition diagram (Figure 2G) showed higher probabilities of transitioning from S1 to W1 (0.07) or W2 (0.07) than from S2 (0.03 and 0.01, respectively). Similarly, probabilities of transitioning from W1 to S1 (0.09) or S2 (0.09) were higher than probabilities of transitioning from W2 (0.04 and 0.01, respectively). Second, we conducted a sleep deprivation experiment where we exposed fish to daytime illumination for the first 6 h of the second night of the experiment (Figure 3A), which has been shown to strongly suppress sleep,^32^ and then turned the lights off. During the remaining 4 h of the night, there was increased sleep (i.e., rebound sleep) compared to the prior night of unperturbed sleep. We then fitted 4-state HMMs to determine how this affected S1 and S2 occupancies and transitions (Figure S6A). Following lights off on the second night, there was a large increase in S2 sleep at the expense of S1 sleep as compared to the first night (Figures 3B and 3C). This suggests that the S2 state may play a crucial role in the homeostatic response to sleep deprivation in zebrafish.

Third, we conducted an experiment where we exposed fish to a mechano-acoustic stimulus every 5 min during the night (Figure 3D). We then fitted a 4-state HMM (Figures 3E and S6B) to classify the sleep state just prior to each stimulus and determined the proportion of fish that showed any activity in the first 2 s after the stimulus, corrected for basal locomotor activity levels (see STAR Methods). The proportion of fish that responded to the stimulus was significantly lower for S2 compared to S1, and for S1 compared to W1 (Figure 3F). Thus, according to all three criteria, S2 represents a deeper sleep state than S1.

The latter experiment also allowed us to test whether HMM state assignments could differentiate arousability even for bins with the same absolute activity level. For this, we exploited the overlap between the activity distributions assigned to S1 and W1 states. In particular, for this experiment, we focused on bins preceding stimuli with activity 5 s/min, 40% of which were classified as S1 and 60% as W1. Remarkably, despite identical activity levels, fish were significantly less likely to respond to the stimulus when the state was classified as S1 compared to W1 (Figure S6C). This further demonstrates the ability of the HMM to extract biologically meaningful underlying states in a way that exceeds the predictive power of simple thresholding.

In the remainder of this work, we used the HMM approach to gain insight into how the disruption of key mechanisms that regulate sleep affects sleep architecture, in particular states S1 and S2. Consistent with previous work using conventional sleep measures, we found no significant differences between +/+ vs. +/− fish for a given mutation (data not shown), so we focused our analyses on +/− vs. −/− mutant comparisons.

### Melatonin deficiency is associated with less deep sleep and more light sleep at night

**Figure 4:**
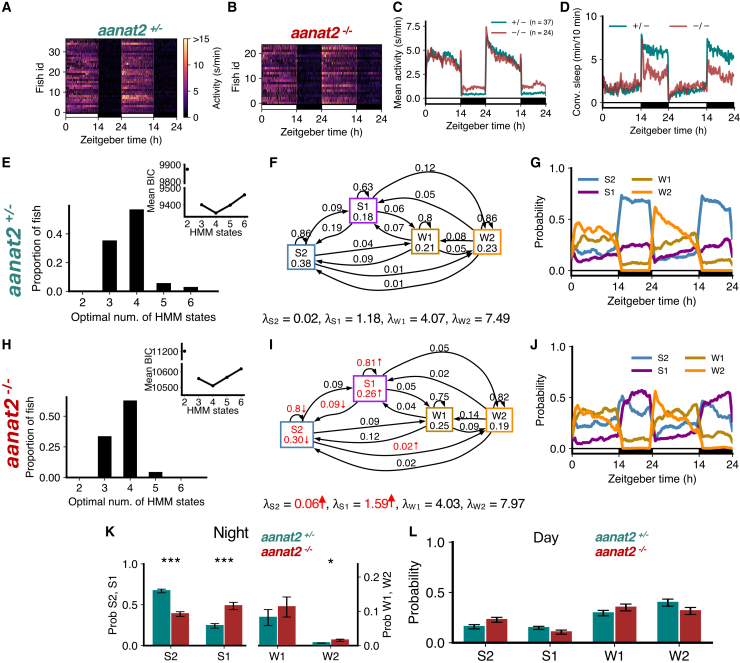
Melatonin-deficient *aanat2* mutants show loss of S2 and gain of S1 (A–D) *aanat2*^−/−^ fish shows higher activity and lower sleep at night than *aanat2*^+/−^ fish. (E and H) Both genotypes were best fit by 4 HMM states. (F and I) *aanat2*^−/−^ fish had several significant differences in fitted HMM parameters (p < 0.05 indicated in red; bootstrap sampling with permutation tests; units of λs: s/min). (G and J) *aanat2*^+/−^ and *aanat2*^−/−^ fish showed strikingly differing patterns of sleep during the night. (K) At night *aanat2*^−/−^ fish had significantly less S2 but significantly more S1. Note the right y axis for states W1 and W2. (L) During the day, there were no significant differences in genotypes between the times spent in each state. Asterisks indicate significant differences (t-tests, with Benjamini-Hochberg multiple comparisons correction; ∗∗∗p<0.001, ∗∗p<0.01, ∗p<0.05). In K, L, data are represented as mean ± SEM. In A–D, G, and J, the white and black horizontal bars depict the lights on and lights off periods, respectively.

Previous studies showed that melatonin acts downstream of the circadian clock to mediate the circadian regulation of sleep in zebrafish.^33^ Melatonin is synthesized in the pineal gland at night due to the circadian expression of *aanat2*, which catalyzes the rate-limiting step of melatonin synthesis. Thus, melatonin-deficient *aanat2* mutant zebrafish can be used to study sleep architecture in the absence of circadian regulation of sleep. We first analyzed sleep architecture under standard 14:10 h light-dark conditions, in which *aanat2*^−/−^ fish were more active (Figures 4A–4C) and slept less at night (Figure 4D) than their *aanat2*^+/−^ siblings.^33^

For both *aanat2*^+/−^ and *aanat2*^−/−^ fish, the optimal number of HMM states was 4 (Figures 4E and 4H). Similar to WT fish (Figure 2), sleep at night in *aanat2*^+/−^ fish was dominated by S2 (Figure 4G). However in *aanat2*^−/−^ fish the amount of S2 was substantially reduced during the night (Figures 4G, 4J, 4K, and S7). Surprisingly, the amount of S1 increased (Figures 4G, 4J, and 4K). The proportions of W1 and W2 were relatively unaffected, and there were no changes in any state probabilities during the day (Figure 4L), as expected since little or no melatonin is produced during the day.^33^ For *aanat2*^−/−^ fish there was a significant increase in $\lambda_{S2}$ (i.e., the mean locomotor activity while in the S2 state), a significant decrease in the probability of occupying and remaining in S2 (Figures 4F and 4I), and a significant decrease in the probability of transitioning from S1 to S2. There was also a significant increase in $\lambda_{S1}$ and the probabilities of occupying and remaining in S1 for *aanat2*^−/−^ fish (Figures 4F and 4I). The reduction in S2 is consistent with conventional sleep analysis that *aanat2*^−/−^ fish sleep less at night, but the model suggests this is mostly offset by an increase in S1 rather than wake states.

### Loss of circadian regulation of sleep in free-running *aanat2* mutant fish is due to the loss of circadian oscillation of deep sleep

In order to analyze the circadian regulation of behavior, it is necessary to first entrain animals in light:dark cycles, and then transfer them to constant dark, known as “free-running” conditions. Under these conditions, entrained molecular and behavioral circadian oscillations persist for several days, allowing sleep to be studied without the masking effect of light on behavior.^34^ Using this approach, the circadian regulation of sleep is abolished in *aanat2* mutant zebrafish.^33^

**Figure 5:**
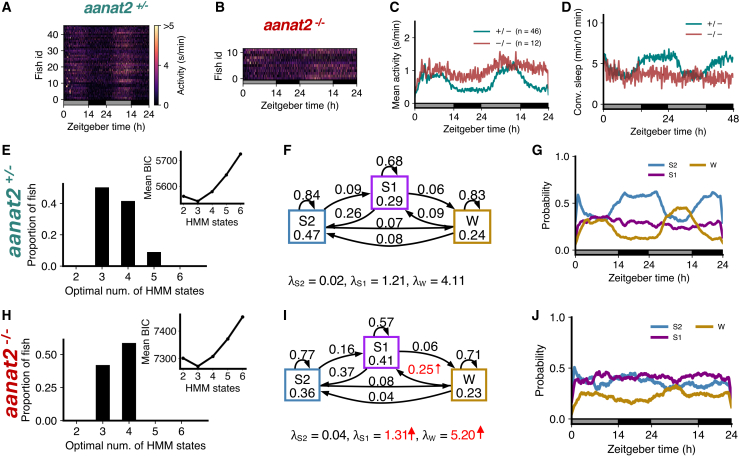
Circadian regulation of sleep at night is due to melatonin-dependent S2 (A–D) Overall activity was reduced under constant darkness, but *aanat2*^−/−^ fish showed higher activity and lower sleep, and lacked circadian oscillations of sleep, at night compared to *aanat2*^+/−^ fish. (E and H) Both genotypes had a minimum BIC at 3 HMM states. (F and I) *aanat2*^−/−^ fish showed significant changes in fitted HMM parameters (p < 0.05 in red; bootstrap sampling with permutation tests; units of λs: s/min). (G and J) *aanat2*^+/−^ fish maintained circadian variation in S2 and W states, but *aanat2*^−/−^ lost circadian variation in all states. In A–D, G, and J, the gray and black horizontal bars depict the subjective day and subjective night periods, respectively.

For both *aanat2*^+/−^ and *aanat2*^−/−^ fish, overall activity levels were reduced in constant dark (Figures 5A–5C), as expected due to the loss of light-induced arousal. However during the subjective night, *aanat2*^−/−^ fish had more activity and less sleep than *aanat2*^+/−^ fish (Figures 5C and 5D). Interestingly, the optimal number of states for the HMM fit of *aanat2*^+/−^ was now only 3 (Figures 5E and 5F). The lowest-activity state λ was similar to $\lambda_{S2}$ for the unperturbed WT case (Figure 2B), and this state showed an increase in probability during subjective night (Figure 5G); we therefore assigned this state as S2. The highest-activity state had a λ similar to W1 for the unperturbed WT case (Figure 2B); this state also showed an increase in probability during the subjective day and we therefore labeled this state as W. The intermediate-activity state showed no obvious variation with subjective day and night; however, its λ was similar to $\lambda_{S1}$ for the unperturbed WT case (Figure 2B), and so we assigned this state as S1. Thus, constant dark has the effect of reducing the number of distinct sleep/wake states by effectively eliminating W2. This implies that the arousing effect of light is required for the W2 state.

For *aanat2*^−/−^ fish, the optimal number of states as defined by the mean BIC over fish was also 3 (Figure 5H). There was a slightly higher number of individual fish with an optimum at 4 states (7 fish) rather than 3 states (5 fish), but for ease of comparison with *aanat2*^+/−^ fish, we analyzed 3-state fits. These fish had significantly higher $\lambda_{S1}$ and $\lambda_{W}$ than *aanat2*^+/−^ Figures 5F and 5I), consistent with generally higher activity levels (Figure 5C). They also showed a significantly higher transition probability from W to S1. However, none of these states showed any circadian variation Figure 5J), demonstrating that the circadian regulation of S2 is abolished by loss of melatonin. Based on these results, we conclude that melatonin mediates the circadian regulation of sleep by increasing the S2 state at night.

### Exogenous melatonin suppresses W2 during the day

**Figure 6:**
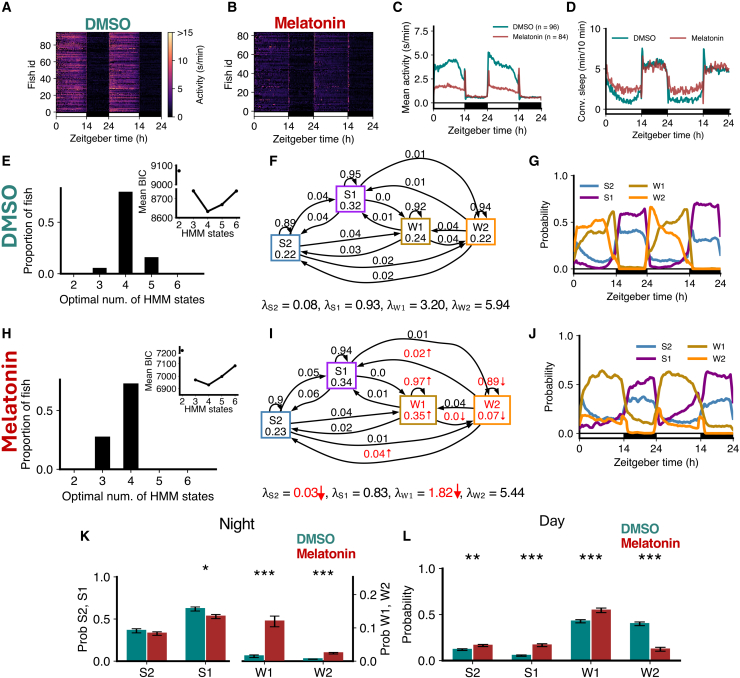
Melatonin-treated fish show loss of W2 during the day (A–D) Exogenous melatonin caused lower activity and higher sleep during the day than control (DMSO-treated) fish. (E and H) Both groups were best fit by 4 HMM states. (F and I) Melatonin-treated fish had several significant differences in fitted HMM parameters (p < 0.05 indicated in red; bootstrap sampling with permutation tests; units of λs: s/min). (G and J) Melatonin fish showed less W2 during the day, with an increase in W1 at night. (K) At night, melatonin-treated fish had significantly less S1 but significantly more W1 and W2. Note the right y axis for states W1 and W2. (L) During the day, W2 significantly decreased for melatonin-treated fish, while S2, S1, and W1 increased. Asterisks indicate significant differences (t-tests, with Benjamini-Hochberg multiple comparisons correction; ∗∗∗p<0.001, ∗∗p<0.01, ∗p<0.05). In K and L, data are represented as mean ± SEM. In A–D, G, and J, the white and black horizontal bars depict the lights on and lights off periods, respectively.

Exogenous melatonin has sleep-promoting effects on larval zebrafish.^26^^,^^35^ To understand how exogenous melatonin impacts sleep and wake sub-states, we compared the activity of WT fish treated with melatonin to DMSO vehicle-treated controls. At night, activity and conventionally defined sleep were unchanged, but during the day, melatonin-treated fish showed decreased activity and increased sleep (Figures 6A–6D). The optimal number of HMM states was 4 for both groups (Figures 6E and 6H). Melatonin-treated fish showed increased occupancy of the W1 state and reduced occupancy of the W2 state (Figures 6F–6I).

The λ values for S2 and W1 states were also lower for melatonin-treated fish (Figures 6F and 6I). During the day, melatonin-treated fish showed an increase in the amount of S2, S1, and W1 at the expense of a large drop in the amount of W2 (Figure 6L).

At night, surprisingly, melatonin-treated fish showed a reduction in S1 and increases in W1 and W2 compared to controls, although a majority of time was still spent in S1 and S2 (Figure 6K). Together, these results show that melatonin suppresses W2 during the day but increases wake states at night. A potential explanation for the latter observation is that, since exogenous melatonin induces sleep during the day, there is less accumulation of homeostatic sleep pressure during the day, and thus less homeostatically driven sleep at night. Endogenous melatonin is present at night, and so exogenous melatonin does not increase sleep at that time.

### Fish that lack serotonin in the raphe nuclei exhibit less deep sleep and altered sleep structure

**Figure 7:**
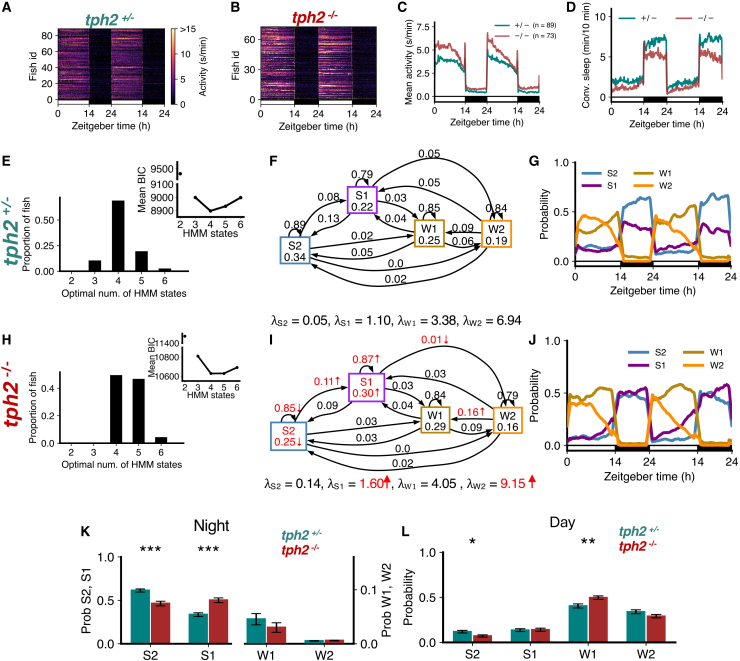
*tph2* mutants show less deep sleep (A–D) *tph2*^−/−^ fish showed higher activity and lower sleep than *tph2*^+/−^ fish. (E and H) Both genotypes were best fit by 4 HMM states. (F and I) *tph2*^−/−^ fish had several significant differences (p < 0.05 indicated in red; bootstrap sampling with permutation tests) in fitted HMM parameters (λ units: s/min). (G and J) *tph2*^+/−^ and *tph2*^−/−^ fish show similar patterns of sleep states during the day but differing patterns at night. (K) At night *tph2*^−/−^ fish had significantly less S2 but significantly more S1. (L) During the day, there were no significant differences in genotypes between the times spent in each state. Asterisks indicate significant differences (t-tests, with Benjamini-Hochberg multiple comparisons correction; ∗∗∗p<0.001, ∗∗p<0.01, ∗p<0.05). In K and L, data are represented as mean ± SEM.

The serotonergic raphe nuclei play an important role in the homeostatic regulation of sleep in both zebrafish and mice.^32^ *tph2* mutant zebrafish, whose raphe nuclei do not synthesize serotonin, are more active than sibling controls (Figures 7A–7C), sleep less (Figure 7D),^32^ and show reduced rebound sleep following sleep deprivation.^32^ Thus, *tph2* mutants can be used to examine sleep architecture in the context of reduced homeostatic sleep pressure. We found that the optimal number of states that fit the data was 4 for both *tph2*^+/−^ controls (Figure 7E) and their *tph2*^−/−^ siblings (Figure 7H). However *tph2*^−/−^ fish had larger λ values for all 4 states, with significant increases for S1 and W2 (Figures 7F and 7I). Additionally, *tph2* mutants had a lower probability of remaining in S2, lower occupancy of S2, and a higher probability of transitioning from S2 to S1 (Figures 7F and 7I). *tph2* mutants also had higher probability of remaining in S1, and higher occupancy of S1 state (Figures 7F and 7I). The S2 amount decreased, and the W1 amount increased during the day (Figures 7G, 7J, and 7L). At night, there was a significant reduction in the proportion of S2, and an increase in S1, in *tph2*^−/−^ fish (Figures 7G, 7J, and 7K). Thus, while conventional analysis indicates a reduction in sleep in *tph2* mutants, HMM analysis instead indicates a shift in the balance between deep and light sleep. This observation suggests that serotonin plays a key role in promoting deep sleep, consistent with previous findings of lighter sleep in *tph2* mutants,^32^ and with the observation that increased homeostatic sleep pressure results in an increase in deep sleep at the expense of light sleep (Figure 3C).

### Pharmacological activation of serotonin signaling results in deeper sleep

**Figure 8:**
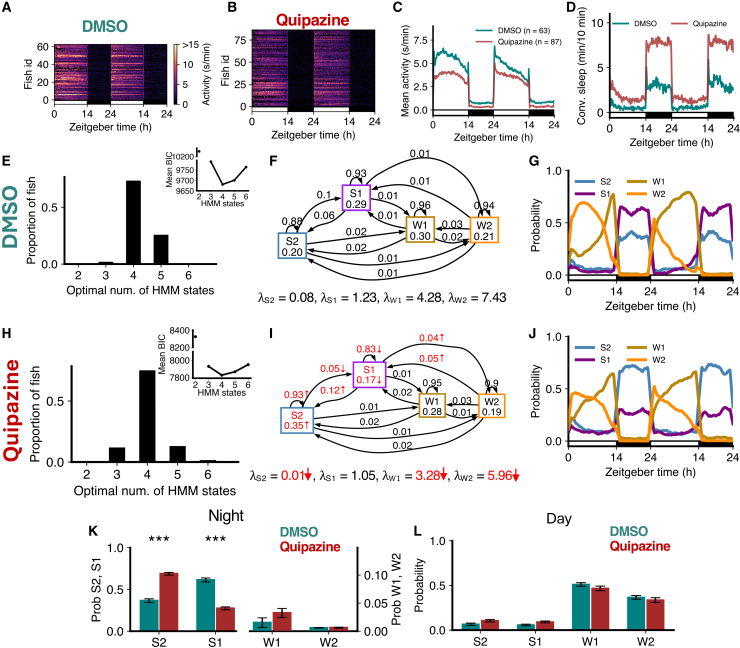
Quipazine treatment induces sleep through an increase in deep sleep (A–D) Quipazine-treated fish showed lower activity and more sleep than DMSO controls. (E and H) Both genotypes were best fit by 4 HMM states. (F and I) Quipazine fish had several significant differences (p < 0.05 indicated in red; bootstrap sampling with permutation tests) in fitted HMM parameters (λ units: s/min). (G and J) Quipazine fish showed changes in sleep states during the night. (K) At night, quipazine fish had significantly higher S2 but significantly lower S1. (L) During the day, there were no significant differences between groups in the times spent in each state. Asterisks indicate significant differences (t-tests, with Benjamini-Hochberg multiple comparisons correction; ∗∗∗p<0.001). In K and L, data are represented as mean ± SEM.

To further explore the role of serotonin in regulating sleep sub-states, we analyzed the behavior of fish treated with quipazine, a serotonin receptor agonist, compared to DMSO vehicle-treated controls. Quipazine treatment results in reduced locomotor activity (Figures 8A–8C) and increased sleep compared to controls (Figure 8D).^32^ For both groups, the optimal number of HMM states was 4 (Figures 8E and 8H). All λ values were reduced (all significantly except $\lambda_{S1}$), consistent with reduced overall activity. The amount of S2 was dramatically increased at night, with a concomitant loss of S1 (Figures 8G, 8J, and 8K), while there were no significant differences during the day (Figure 8L). Consistent with this, there were significant increases in the probabilities of staying in the S2 state and transitioning from S1 to S2, and a decrease in the probability of transitioning from S2 to S1 (Figures 8F and 8I). These results are consistent with the *tph2* mutant data (Figure 7), and with the hypotheses that serotonin promotes deep sleep but not light sleep, and that increased homeostatic sleep pressure promotes deep sleep at the expense of light sleep.

### Noradrenaline-deficient *dbh* mutant fish sleep more due to increased deep sleep

**Figure 9:**
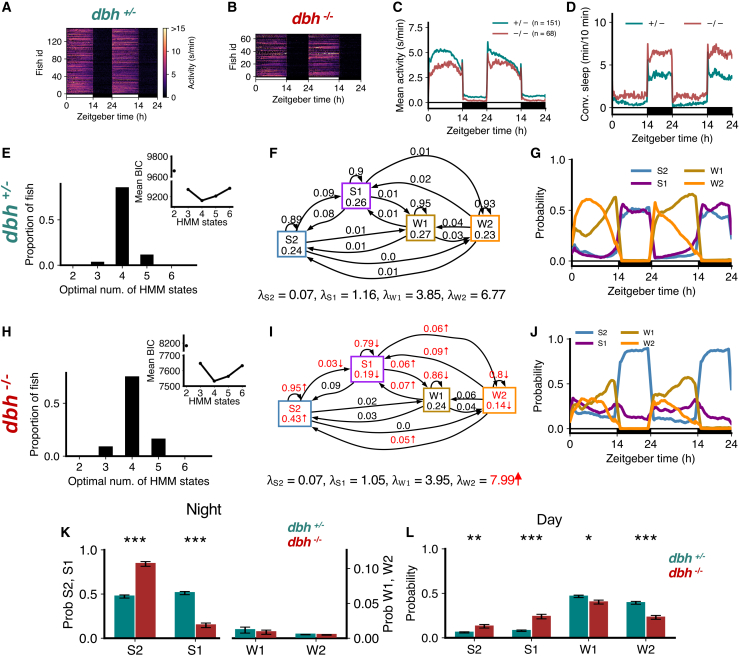
dbh mutants show more deep sleep and less wake occupancy (A–D) *dbh*^−/−^ fish shows lower overall activity and higher sleep than *dbh*^+/−^ fish. (E and H) Both genotypes were best fit by 4 HMM states. (F and I) *dbh*^−/−^ fish had several significant differences (p < 0.05 indicated in red, bootstrap sampling with permutation tests) in fitted HMM parameters (λ units: s/min). (G and J) *dbh*^+/−^ and *dbh*^−/−^ fish show altered patterns of sleep during night and day and wake states during the day. (K) At night, *dbh*^−/−^ fish had significantly high S2 but significantly less S1. (L) During the day, there were significant increases in S2 and S1, and a decrease in W2, in *dbh*^−/−^ fish. Asterisks indicate significant differences (t-tests with Benjamini-Hochberg multiple comparisons correction; ∗∗∗p<0.001, ∗∗p<0.01, ∗p<0.05). In K and L, data are represented as mean ± SEM.

Noradrenaline is a major wake-promoting neurotransmitter that helps maintain arousal and suppresses sleep across vertebrate species, including zebrafish.^36^ *dbh*^−/−^ fish, which lack norepinephrine, were less active (Figures 9A–9C) and slept more (Figure 9D) than sibling *dbh*^+/−^ controls.^36^ Both mutants and controls were best fit by 4 HMM states (Figures 9E and 9H). While there were no significant changes in the λs for S2, S1 and W1, $\lambda_{W2}$ increased significantly in *dbh*^−/−^ fish. *dbh*^−/−^ fish had a significant increase in the occupancy of S2, and a decrease in the probability of transitioning from S2 to S1 (Figures 9F and 9I), leading to their altered proportions during both day and night (Figures 9G and 9J–9L). The probabilities of remaining in wake states dropped significantly (Figures 9F and 9I), resulting in a significant reduction in the proportions of W1 and W2 during the day (Figure 9L). Notably, W2 occupancy and the probability of transitioning from W2 to S2 and S1 increased significantly for *dbh*^−/−^ fish (Figures 9F and 9I). These results suggest that loss of noradrenaline results in increased deep sleep at the expense of light sleep.

### Pharmacological inhibition of noradrenaline signaling phenocopies *dbh* mutant behavior

**Figure 10:**
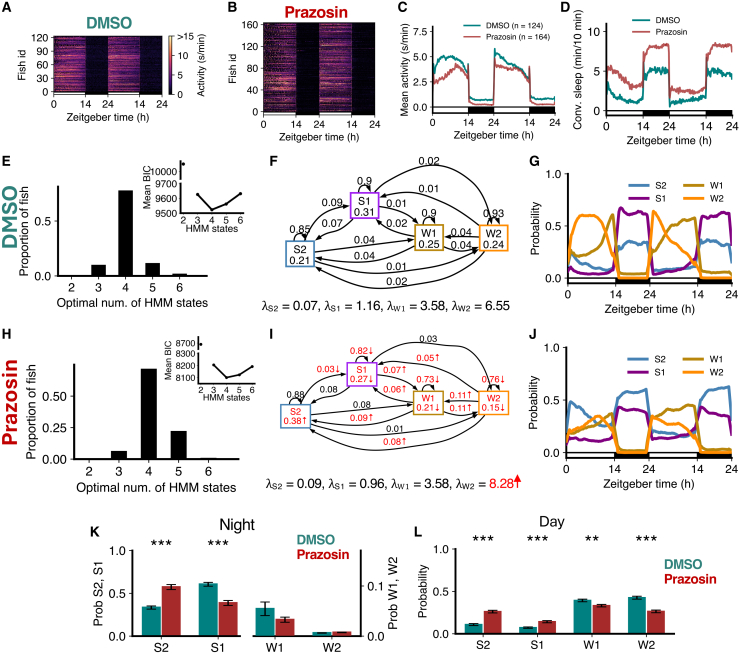
Prazosin treatment induces sleep through gain in deep sleep and a loss of wake (A–D) Prazosin-treated fish showed lower activity and higher sleep than DMSO controls. (E and H) Both groups were best fit by 4 HMM states. (F and I) Prazosin-treated fish had several significant differences (p < 0.05 indicated in red, bootstrap sampling with permutation tests) in fitted HMM parameters (λ units: s/min). (G and J) Prazosin fish show an increase in S2 during the night and day, and reduced W2 during the day. (K) At night, prazosin fish showed a significant increase in S2 and a significant decrease in S1. (L) During the day, there were significant differences between groups in the times spent in each of S2, S1, and W2. Asterisks indicate significant differences (t-tests with Benjamini-Hochberg multiple comparisons correction; ∗∗∗p<0.001, ∗∗p<0.01, ∗p<0.05). In K and L, data are represented as mean ± SEM.

Prazosin is an alpha1-adrenergic receptor antagonist, which blocks the postsynaptic effects of noradrenaline alpha1 receptors, and thus phenocopies *dbh* mutant fish.^36^ Prazosin-treated fish had lower levels of locomotor activity (Figures 10A–10C) and slept more at night than controls (Figure 10D).^36^ In both cases, the optimal number of HMM states was 4 (Figures 10E and 10H). In the HMM transition diagram, there was a significant increase in the occupancy of S2, a decrease in the occupancies of S1, W1, and W2, accompanied by increases in the probability of transitioning from W2 to lower activity states, and a decrease in the probability of remaining in S1, W1, and W2 (Figures 10F and 10I).

There were significant changes in the amounts of S1 and S2 both during night and day in the drug-treated fish (Figures 10G and 10J–10L), indicating that the S2 occupancy change was also driven by daytime changes. Consistent with this, during the day, there was a large increase in the proportion of S2 and S1 and a decrease in the proportion of W1 and W2 (Figure 10L). Consistent with the *dbh*^−/−^ fish, prazosin-treated fish did not show significant differences in λs except for the W2 state (Figures 10F and 10I). Since noradrenaline signaling normally promotes arousal during the day, this suggests that alpha1-adrenergic tone suppresses transitions into deep sleep during the active phase. These results support a model in which noradrenaline, via alpha1 receptors, promotes wakefulness by limiting inappropriate or premature entry into deep sleep when animals would otherwise be active. Compared with *dbh* mutant fish (Figures 9K and 9L), prazosin-treated fish showed similar changes in states both during the night and day, consistent with the established role of noradrenaline signaling.

## Discussion

Although recent computational analyses of freely moving zebrafish have shed light on the temporal organization of behavior,^37^^,^^38^^,^^39^^,^^40^^,^^41^ none have specifically addressed sleep. Here, by applying HMMs to long-term locomotor recordings, we reveal that zebrafish sleep comprises two distinct sub-states. These states occur predominantly at night, display distinct timing, transition dynamics, arousability, and rebound following sleep deprivation, and are differentially modulated by neuromodulatory systems. This richer characterization aligns with evidence from other species that sleep comprises multiple functional sub-states rather than a unitary condition. By establishing a robust computational framework for dissecting sleep architecture in larval zebrafish, this work paves the way for further insights into sleep regulation.

Traditional definitions of zebrafish sleep, based on quiescence thresholds, are primarily captured by S2, with $\lambda_{S2}\approx$ 0 s/min (Figure 1I). The S1 state is characterized by non-zero but much lower levels of activity than wake states ($\lambda_{S1}\approx$ 1 s/min, as compared to $\lambda_{W1}\approx$ 4 s/min and $\lambda_{W2}\approx$ 8 s/min). The statistically derived value of $\lambda_{S1}\approx$ 1 s/min matches with an earlier analysis, which heuristically specified a 3-state model where between 0 and 1 s/min of activity was defined as a “low-activity” state and more than 1 s/min activity as a “high-activity” state.^25^ While S1 generally occurs more at night, it shows the surprising property of being increased by manipulations that reduce total sleep, defined conventionally in zebrafish. Indeed, changes in S1 often appear to compensate for changes in S2. This follows naturally from the structure of the HMM model: reductions in S2 caused by a small increase in overall activity will mostly be captured by S1, since the activity increase required to be captured by the W states is larger.

The S1 state’s arousal threshold clearly distinguishes it from either wake state (Figure 3F). However, its intermediate responsiveness compared to S2 and more frequent transitions to wake states (Figure 2), suggest that it could represent a shallow, transitional sleep state rather than a stable, deep sleep stage. Functionally, S1 may act as a gateway into or out of deep sleep, or as a default idling state when sleep pressure is low, and conditions do not favor the deeper, more quiescent S2 state. The observation that *aanat2−/−* and *tph2−/−* mutants show decreased S2 accompanied by increased S1 supports the idea that these neuromodulatory pathways promote consolidation into deeper S2 sleep, while S1 may persist when this drive is weakened, suggesting that S1 represents an inability to achieve or maintain the deeper S2 state. In mammals, serotonin and melatonin promote entry into and maintenance of NREM sleep,^32^^,^^42^ whereas noradrenaline promotes arousal and opposes sleep.^43^ Our findings that the activation of serotonin signaling (due to quipazine treatment) and inhibition of noradrenergic signaling (in *dbh mutants* and due to prazosin treatment) increase S2, while loss of melatonin signaling (in *aanat2* mutants) results in decreased S2, suggest that these pathways primarily impact sleep by controlling the amount of time spent in the S2 deep sleep state.

The precise nature of the S1 state could potentially be addressed by higher-resolution analysis of more subtle behaviors, as well as measures of physiology, though these experiments would be much lower throughput than the simple locomotor activity data we have analyzed here. Ultimately, electrophysiological recordings will be required to fully determine how S1 and S2 states compare with standard definitions of mammalian sleep states. Leung et al.^23^ recorded calcium activity in the dorsal pallium of head-restrained zebrafish larvae using 1-photon microscopy and reported states somewhat analogous to NREM and REM, the latter of which they termed propagating wave sleep. However, these states were only observed after prolonged sleep deprivation, perhaps because natural sleep was inhibited by the requirement to restrain the animals for imaging, or by the intense visible light required to excite GCaMP fluorescence. Our data suggest that zebrafish S1 and S2 states are more analogous to light and deep stages of NREM sleep in mammals than REM and NREM, respectively, although higher resolution behavioral data and neuronal activity monitoring in a manner that does not use visible excitation light are needed to fully address this question.

HMMs have emerged as a powerful computational framework for analyzing sleep architecture across diverse species, leveraging their ability to capture the temporal dynamics and probabilistic transitions between distinct behavioral states. In human sleep research, HMMs have been extensively applied to automate sleep stage classification from polysomnographic recordings, utilizing EEG, EOG, and EMG signals to identify transitions between sleep stages while accounting for the inherent temporal structure of sleep architecture.^44^^,^^45^ HMMs have also been adapted for sleep/wake identification using human actigraphy data, expanding their utility to large-scale studies where traditional polysomnography is impractical.^46^ In mice, four-state Markov models have been used to characterize sleep-wakefulness dynamics along light/dark cycles.^47^ In invertebrate models, probabilistic analyses including HMM-based approaches have revealed covert sleep-related biological processes in *Drosophila*, enabling the quantification of sleep pressure and sleep depth parameters.^17^ The versatility of HMMs in capturing both observable behavioral patterns and inferring hidden states makes them particularly well-suited for comparative sleep studies across phylogenetically diverse species, providing a unified analytical framework that can accommodate the varied temporal scales and behavioral manifestations of sleep across different animal models. In our work, we used BIC to determine the optimal number of states and were careful to address the non-deterministic nature of HMM fitting, fitting the model multiple times to each fish and then choosing the one with the highest log likelihood.

Our HMM approach infers hidden states based solely on statistical regularities in the behavioral data, without guaranteeing that the inferred states correspond directly to biologically meaningful categories. However, previous work has demonstrated that HMMs applied purely to human actigraphy data can accurately reproduce sleep states derived from simultaneous polysomnography.^46^ Moving beyond sleep, there is compelling evidence that HMMs applied to mouse behavioral data can reveal hidden states corresponding to known physiological categories.^48^ This work demonstrates the power of behaviorally based approaches for revealing physiological states, especially when physiological measurements are hard to obtain directly.

Our modeling approach also revealed individual variability in sleep state architecture. While four was always the modal optimal number of states (except in free-running conditions when the W2 state was lost), in each condition, some fish showed three, five, or even six states. However, two states were never the optimal solution, showing that a binary classification into unitary “sleep” and “wake” states is an incomplete characterization of zebrafish sleep-wake cycles. The variation in the number of optimal states that we observed between individual fish is not surprising, given the documented individual differences in sleep/wake behaviors in fish^25^^,^^26^^,^^49^ and mammals, including humans.^50^ This variability could arise from multiple sources, including developmental stochasticity in neural circuit formation,^51^ subtle microenvironmental variation during early development, and epigenetic differences. This mirrors well-documented individual differences in human sleep architecture, chronotype, and sleep need, which persist over time and are only partially explained by genetic variation.

We also observed variability in the proportions of S1 and S2 between different control groups. Although for each experiment the mutant/drug case was performed at the same time as the controls, we have collectively analyzed data collected over many years. This long-term variability likely reflects a combination of factors, including individual differences, subtle environmental fluctuations, and changes in video-tracking technology. Consider the case of a small decrease d in the amount of conventionally defined sleep between two experiments being the result of a small increment in overall activity. Time bins lost to S2 will be captured mostly by S1, so that the S2:S1 ratio is expected to change by approximately (1-d)/(1+d) ∼ 1-2d, i.e., a change twice as large as the change in conventionally defined sleep. However, the directional shifts we observed in sleep states were consistent across multiple independent manipulations targeting distinct neuromodulatory pathways, each compared with its respective control group. This consistency suggests that the effects we report reflect robust changes in sleep architecture rather than sampling noise. A potentially important direction for future work is to reduce batch variability by running multiple manipulations in parallel with shared controls.

By examining how specific genetic and pharmacological perturbations alter the occupancy and transitions of sleep states, we demonstrate that distinct neuromodulators play complementary roles in shaping zebrafish sleep architecture. Melatonin acts downstream of the circadian clock to support deeper sleep at night, emphasizing the importance of circadian output for sleep depth as well as timing. Serotonin appears critical for promoting progression into and maintenance of deep sleep: loss of serotonin in *tph2* mutants reduces S2, while the serotonin agonist quipazine shifts sleep in the opposite direction. Loss of noradrenaline increases deep sleep and decreases maintenance of wakefulness during the day. In contrast, at night, noradrenaline-deficient mutants exhibit increased deep sleep and reduced light sleep. Together, these findings show that monoaminergic neuromodulators do not simply gate sleep-wake transitions but actively shape the internal architecture of sleep, biasing the balance between lighter and deeper sleep sub-states with time of day.

Together, our results suggest that the ability to structure sleep into depth-defined sub-states is an evolutionarily conserved feature that does not depend on mammalian-specific brain structures. More broadly, our work highlights the importance of analyzing sleep microarchitecture to uncover how neuromodulatory pathways shape not just when animals sleep, but how deeply they sleep. This provides a more nuanced view of sleep regulation that bridges behavior, circuits, and evolution.

### Limitations of the study

Several limitations should be noted. First, as described above, our analysis is based solely on locomotor activity, without simultaneous neural recordings to determine the neural signatures of the states we have defined behaviorally. Second, the HMM framework imposes assumptions such as the Markov property and that activity within each state follows a Poisson distribution. While these assumptions enable tractable inference and the model fits the data well, the underlying biology likely involves longer temporal dependencies. Third, the data analyzed here did not include other potentially informative features such as body posture, eye movements, fin movements, or heart rate. These additional measures could potentially enable finer-grained state classifications. Fourth, our experiments used 5–6 dpf larval zebrafish in individual wells under controlled laboratory conditions with a fixed 14:10 h light-dark cycle. Whether these sleep sub-states persist into adulthood and differ in more naturalistic environmental conditions, including social interactions, remains to be determined. Lastly, these data were collected over several years with separate control groups for each experimental comparison. While this ensures appropriately matched controls for each manipulation, it introduces batch-to-batch variability in absolute state proportions. However, despite this variability, the consistency of directional effects across multiple independent manipulations supports the robustness of our findings.

## Resource availability

### Lead contact

Requests for further information and resources should be directed to and will be fulfilled by the lead contact, Geoffrey Goodhill (g.goodhill@wustl.edu).

### Materials availability

This study did not generate any new material.

### Data and code availability

• Data: Behavioral datasets used in this work have been deposited at Figshare [https://doi.org/10.6084/m9.figshare.30727589].
• Code: All original code used for Hidden Markov Model fitting, state inference, and figure generation reported in this article has been deposited at Figshare [https://doi.org/10.6084/m9.figshare.30727589].
• Any additional information required to reanalyze the data reported in this article are available from the lead contact upon request.

## Acknowledgments

This work was supported by the 10.13039/100000002NIH grants UF1 NS126562 to D.A.P and G.J.G, R35 NS122172-04S1 to O.E., and R35 NS122172 to D.A.P. We thank Bruno Van Swinderen and Maxwell Shafer for valuable feedback on an earlier version of the article.

## Author contributions

Conceptualization and experimental design, R.T., G.J.G., and D.A.P.; behavioral experiments, G.O. and O.E.; Python code and implementation, R.T.; writing – original draft, R.T., G.J.G.; writing – review and editing, R.T., G.O., G.J.G., and D.A.P.; funding acquisition, D.A.P. and G.J.G.; supervision, G.J.G.

## Declaration of interests

The authors declare no competing interests.

## STAR★Methods

### Key resources table

**Table:** 

REAGENT or RESOURCE	SOURCE	IDENTIFIER
DMSO	VWR	CAT# MK49494802
Melatonin	Sigma	CAT# M5250
Quipazine maleate	Sigma	CAT# Q1004
Prazosin hydrochloride	Sigma–Aldrich	P7791

**Behavioral assays**		

Behavioral Assays	Gandhi et al.^33^	N/A

**Deposited data**		

Behavioral recordings and code	This paper	Figshare: https://doi.org/10.6084/m9.figshare.30727589

**Experimental models: Organisms/strains**		

Zebrafish AB strain	Zebrafish International Resource Center	ZDB-GENO-960809-7
Zebrafish TL strain	Zebrafish International Resource Center	ZDB-GENO-990623-2
*aanat2-/- ct801*	Gandhi et al.^33^	ZDB-ALT-131122-2
*tph2-/- ct817*	Oikonomou et al.^32^	ZDB-ALT-131122-14
*dbh-/- ct806*	Singh et al.^36^	ZDB-ALT-131122-7

**Software and algorithms**		

Python (version 3.11)	Python Software Foundation	https://www.python.org/
Zebrabox	Viewpoint LifeSciences	N/A

### Experimental model and study participant details

#### Zebrafish experiments

Animal husbandry and all experimental procedures involving animals were performed in accordance with the California Institute of Technology Institutional Animal Care and Use Committee (IACUC) guidelines and by the Office of Laboratory Animal Resources at the California Institute of Technology (animal protocol 1580). Zebrafish were raised and maintained at 28.5°C under 14:10 hours light:dark conditions with white lights on from 9 a.m. to 11 p.m. At 4 dpf, individual zebrafish were placed in each well of a 96-well plate, and locomotor activity and sleep were monitored for 48 h from 5–6 dpf, starting with lights on at 9 a.m. at 5 dpf, using a videotracking system as previously described.^52^ Although the larvae could potentially see other larvae in neighboring wells, zebrafish do not display social behaviors until about 3 weeks of age (^53^^,^^54^). Behavior was monitored at 30 Hz and data were integrated into 1-minute bins for the HMM analyses. Details on fish husbandry, matings, and genotyping can be found in previous papers that presented these datasets.^32^^,^^33^^,^^36^ Sleep deprivation and acoustic stimulation were performed as described.^32^ For the experiment with exogeneous melatonin treatment, we treated fish with 10 μ M melatonin dissolved in 0.1% DMSO or 0.1% DMSO vehicle control and performed behavioral analysis as previously described.^33^ In the sleep deprivation experiment, during the second night of behavioral recording, the white lights that normally turned off at 11 p.m. were kept on for 6 hours until 5 a.m., and then turned off for 4 hours until 9 a.m. In the acoustic stimulus experiment, a stimulus was delivered every 5 minutes from 12:30 a.m. to 9 a.m. in the dark while behavior was monitored (99 stimuli total).

### Method details

#### HMM fitting

We used Poisson HMMs (PoissonHMM module of the hmmlearn library in Python) to fit the fish activity, which was quantified by the number of active seconds in each 60-second bin. For BIC calculations, we fitted 1000 models with different random seeds for each fish for each of 2–6 states and selected the model in each case with the maximum log-likelihood. For each fish, state occupancies were estimated using the dominant eigenvector of the transition probability matrix. Average state probabilities over time were smoothed with a window of 60 minutes.

For the sleep deprivation and arousal experiments, only 4-state HMMs were fitted (again the best of 1000 fits). For the arousal experiment the data per fish spanned only a single night (10 h), which provides less reliable state fitting (Figure S1B). For this case we therefore considered concatenating data from all fish together and then fitting a single 4-state HMM, in addition to individual fits. From the fitted model we inferred state assignments over time for each fish. To allow a fair comparison, we excluded fish that did not occupy each of the four states at least once during the experiment. This left 24 of an initial 42 fish remaining. The 1-minute time bins were aligned so that each stimulus occurred just after the end of a bin. The state assigned to this previous bin was taken as the state of the fish at the time of the stimulus. The fish was recorded as having responded to the stimulus if there was any movement in the 2 seconds following the stimulus. To correct this initial probability for baseline activity (i.e., the probability that a fish in a particular state would have moved in a 2-second window without the stimulus), we applied the same procedure but based on the 1-minute time bin starting 2 minutes before the stimulus, and then subtracted this probability from that calculated initially.

### Quantification and statistical analysis

To test for significant differences between HMM parameters for fish in different groups, we performed bootstrap sampling from the two groups and a permutation test to determine a p-value (with p < 0.05 taken as significant). This procedure was applied for all state occupancies, λs, and transition probabilities. A Bonferroni correction was used for multiple comparisons (a factor of 4 for state occupancies and λs, and 16 for transition probabilities), with significance depicted with ∗∗∗ for p<0.001, ∗∗ for p<0.01, ∗ for p<0.05. For the arousal threshold experiment we used t-tests and Benjamini–Hochberg correction for multiple comparisons to compare response fractions in different states, and also compared with significance levels from with arcsin-transformed response fractions.

### Additional resources

This paper did not generate new websites or online resources. All original code and data used in this study is deposited at Figshare (https://doi.org/10.6084/m9.figshare.30727589).
