# Supplementary material for: Zebrafish sleep displays distinct sub-states
Source: iScience. 2026 Feb 12;29(3):115004. doi: 10.1016/j.isci.2026.115004 (PMC12969347; doi:10.1016/j.isci.2026.115004)
Supplement: Document S1. Figures S1–S7 and Tables S1–S3 [file mmc1.pdf]

iScience, Volume *29*

Supplemental information

Zebrafish sleep displays distinct sub-states

Richa Tripathi, Grigorios Oikonomou, Olivia Eliopoulos, David A. Prober, and Geoffrey J. Goodhill

Document S1. Related to “Zebrafish sleep displays distinct sub-states”

Richa Tripathi[1], Grigorios Oikonomou[2], Olivia Eliopoulos[2], David A. Prober[2], Geoffrey J. Goodhill[1,*]

1 Departments of Developmental Biology and Neuroscience, Washington University in St. Louis, St. Louis, Missouri 63110, USA
2 Tianqiao and Chrissy Chen Institute for Neuroscience, Division of Biology and Biological Engineering, California Institute of Technology, Pasadena, CA 91125, USA
***** Corresponding author, g.goodhill@wustl.edu

Supplementary Table 1. λ values for fish with 4 states as optimal

	λ_{S2}	λ_{S1}	λ_{W1}	λ_{W2}
fish 1	0.02	1.27	4.65	7.04
fish 2	0.01	0.23	0.91	5.89
fish 3	0.01	1.10	5.76	8.23
fish 7	0.01	1.33	3.67	8.97
fish 9	0.01	1.00	4.97	8.86
fish 10	0.00	1.27	4.18	5.87
fish 11	0.02	1.16	3.85	7.08
fish 18	0.02	0.94	3.17	8.52
fish 19	0.02	1.35	5.72	8.70

Supplementary Table 2. λ values for fish with 3 states as optimal

fish 4 (3 state)	0.02	1.55	7.00	
fish 4 (4 state)	0.02	1.29	5.50	8.65
fish 15 (3 state)	0.03	1.79	8.91	
fish 15 (4 state)	0.03	1.26	2.23	9.06
fish 17 (3 state)	0.01	2.15	7.24	
fish 17 (4 state)	0.00	1.21	4.01	7.91
fish 20 (3 state)	0.01	1.52	4.77	
fish 20 (4 state)	0.00	1.06	3.41	5.87

Supplementary Table 3. λ values for fish with 5 states as optimal

fish 5 (5 state)	0.04	1.11	1.16	4.62	6.79
fish 5 (4 state)	0.04	1.16	1.36	5.33	
fish 6 (5 state)	0.04	0.09	1.28	3.71	5.12
fish 6 (4 state)	0.05	1.49	3.92	5.13	
fish 8 (5 state)	0.01	0.11	1.53	3.14	5.30
fish 8 (4 state)	0.01	0.31	1.54	4.28	
fish 12 (5 state)	0.03	0.40	1.23	4.08	7.69
fish 12 (4 state)	0.03	1.23	2.49	6.97	
fish 13 (5 state)	0.02	0.18	2.11	3.45	7.62
fish 13 (4 state)	0.04	2.11	6.35	9.56	
fish 14 (5 state)	0.01	0.02	1.29	3.42	7.73
fish 14 (4 state)	0.00	1.36	4.93	8.22	
fish 16 (5 state)	0.03	0.70	1.54	4.62	10.45
fish 16 (4 state)	0.05	1.50	5.27	10.63	

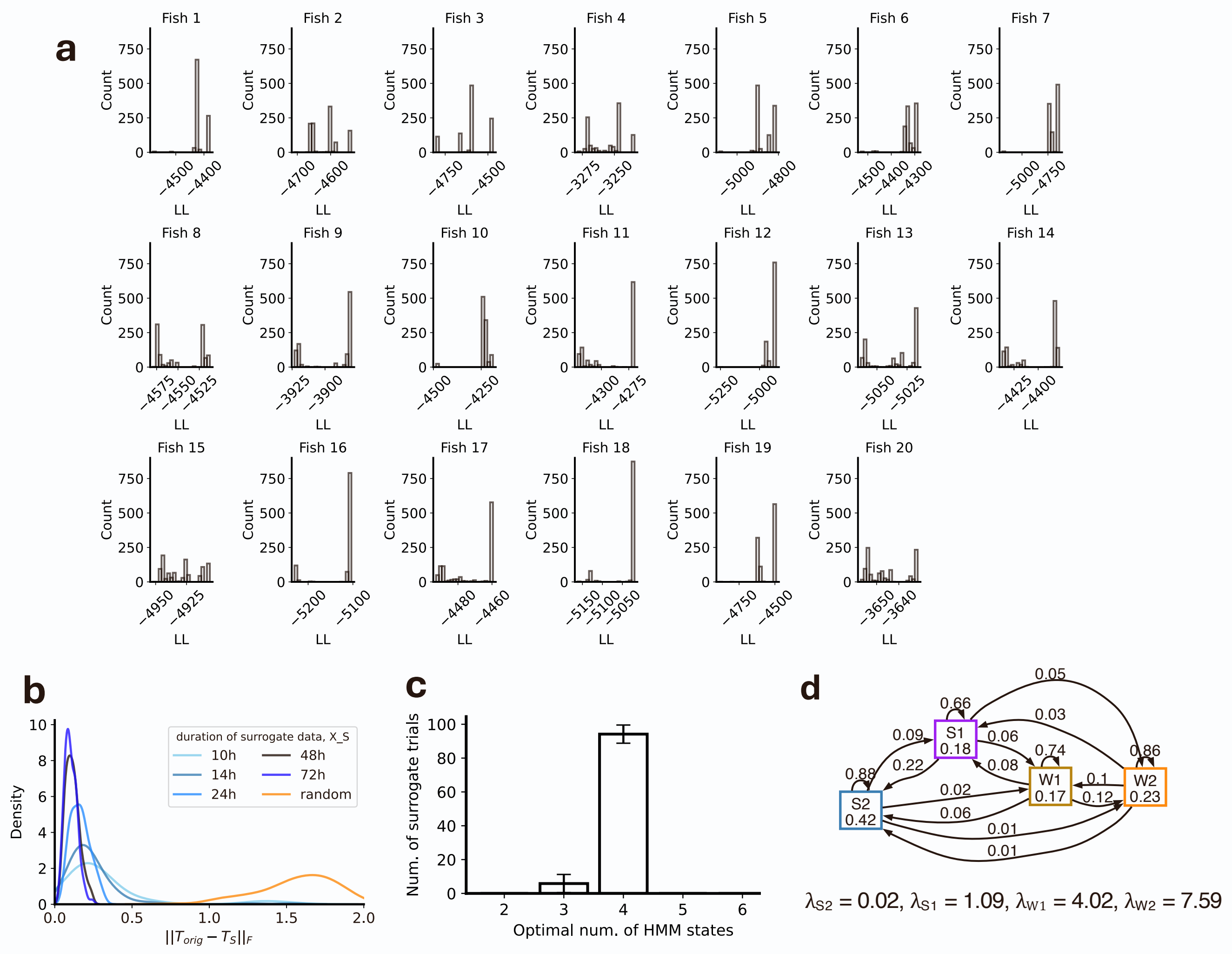

Figure S1: **Robustness of HMM parameter fits.** (**a**) Distribution of log-likelihood (LL) over 1000 model fits for each WT fish, depicting variability among different runs. (**b**) Distribution of difference (Frobenius norm) between the transition probability matrix T_{orig} fitted on 48 h fish data and the transition probability matrix fitted on surrogate data generated from T_{orig}, as a function of the amount of surrogate data provided. The distributions became narrower as longer durations of data were used for fitting, with little change after a duration of 48 h. For the random distribution case each row of the transition probability matrix was generated from a Dirichlet distribution and normalized to sum to one. (**c**) For surrogate data generated 100 times from a 4-HMM fit to a fish for which 4 states was optimal, the optimal number of fitted states was 4 in 94 cases. The error bars are standard error of mean over fish. (**d**) Mean transition diagram and λs from HMM fits to the first 24 h for each fish. There were no significant differences in derived HMM parameters from the fits to 48 h.

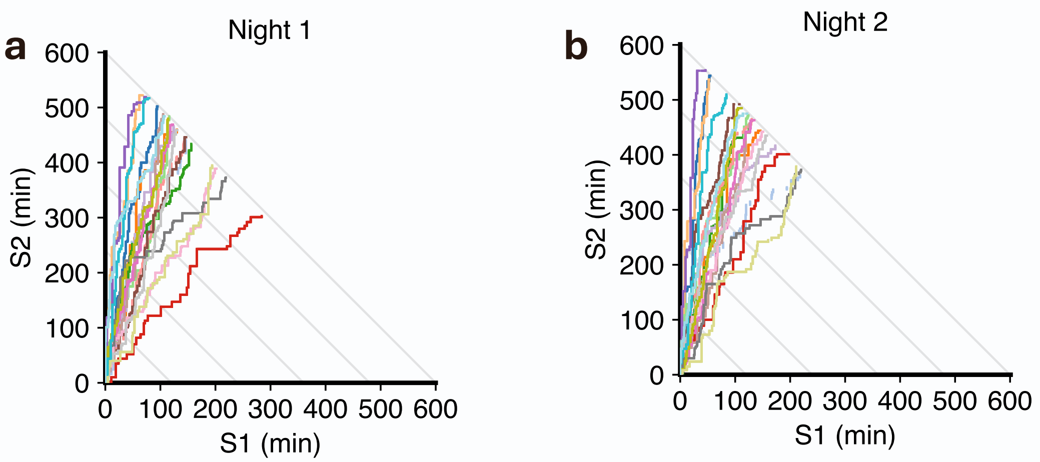

Figure S2: **Complete S1 and S2 state sequences at night for all WT fish.** In this representation night starts at the origin, and then we draw a horizontal line for each minute in state S1 and a vertical line for each minute in S2. Each color corresponds to a different fish (matched between nights). In the rare instances when a wake state occurs we leave a horizontal space. All lines thus finish at 600 total min (10 h). These diagrams provide a compact way of representing individual sleep-state sequences for all fish in the same plot.

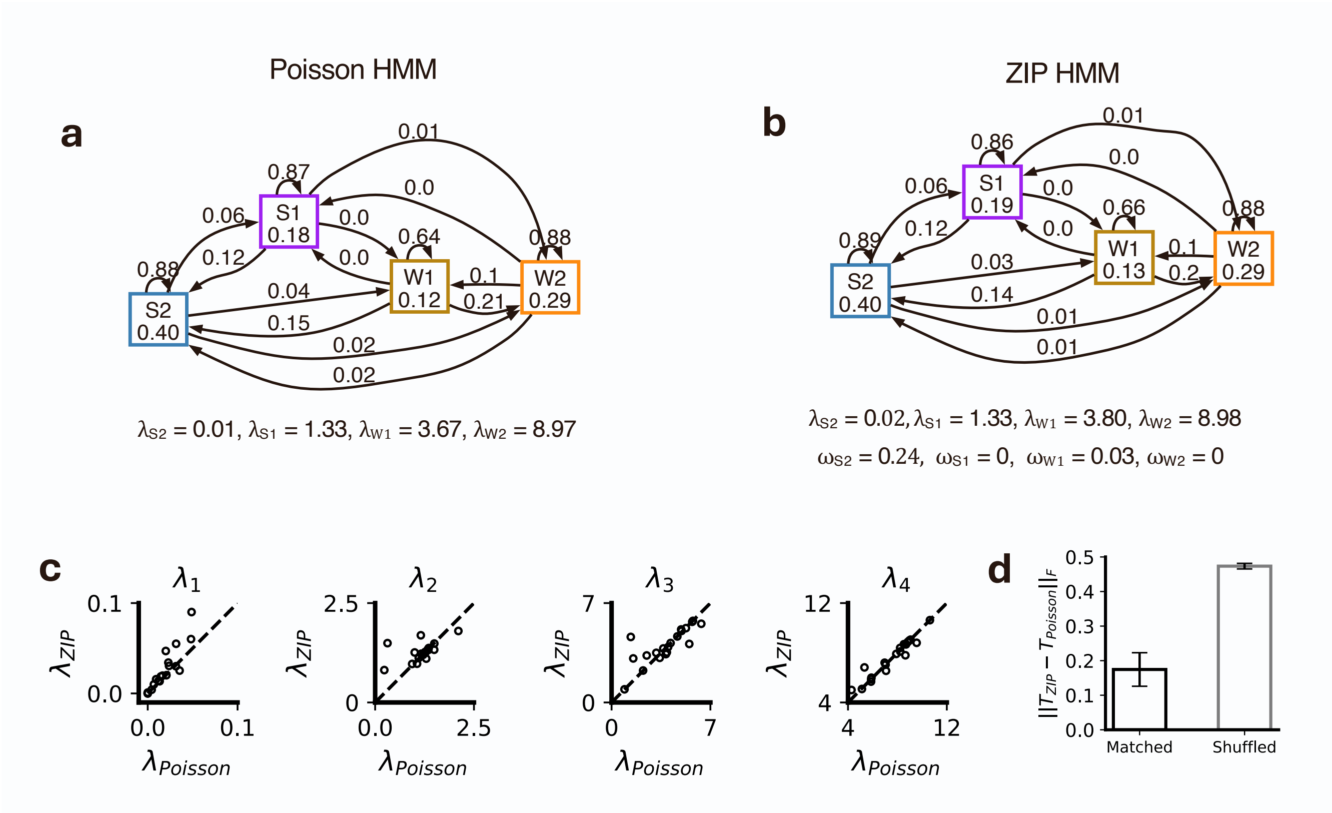

Figure S3: **Comparison of Poisson HMM fits with Zero-Inflated Poisson HMM (ZIP HMM) fits.** (**a**,**b**) Poisson (a) and ZIP (b) transition diagrams for an example fish. In b the zero-inflation probabilities (ω) are also quoted. (**c**) Comparison of fitted λs for all the fish for the two models (one dot per fish). (**d**) The mean Frobenius norm of the difference of transition matrices for each fish for the two models compared to those from shuffled fish pairs.

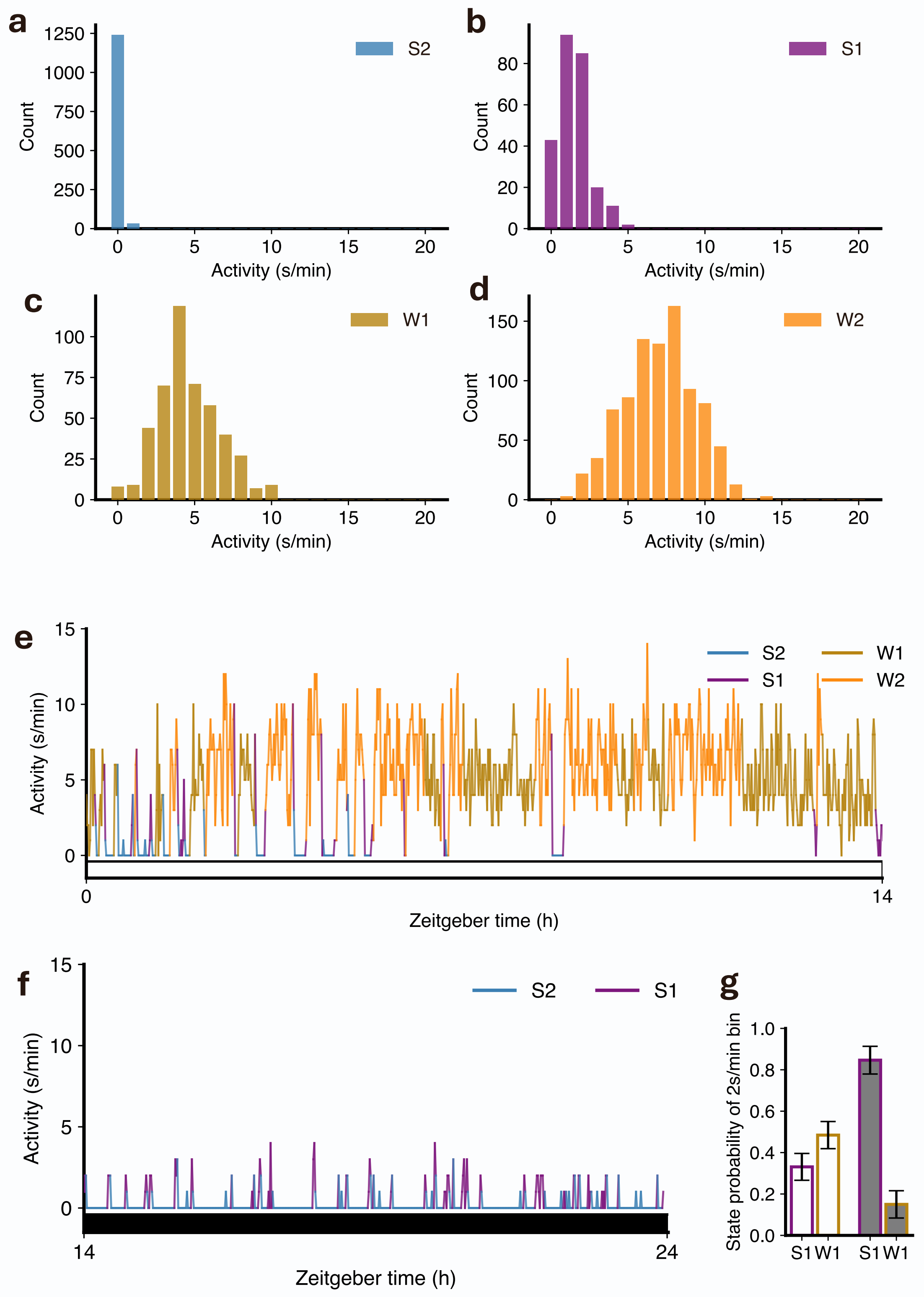

Figure S4: **Relation of activity levels to state assignments and arousal probability.** (**a-d**) Histograms for an example WT fish showing the distribution of activity levels assigned to each of the four states. (**e-f**) Activity levels of the same fish colored by the state assigned at each time during the first day (**e**) and night (**f**). (**g**) On average, bins with 2 s/min of activity are more likely to get assigned state W1 during the day (empty bars), but state S1 during the night (filled bars).

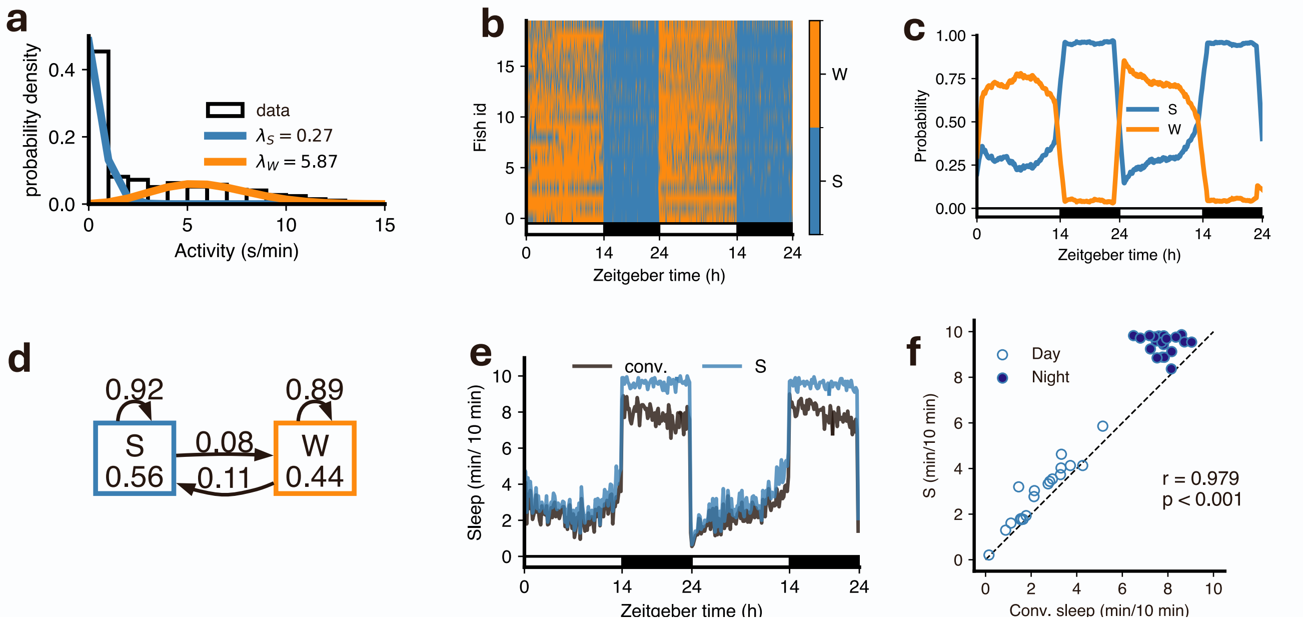

Figure S5: **A 2-HMM fit to WT fish data.** 91(**a**) λs for Poisson distribution fit to activities in both states (S and W). (**b**) Most likely state sequences underlying fish activities. (**c**) State probabilities during day and night phases. (**d**) Transition diagram for the model. (**e**) A comparison of sleep amounts for the S state and sleep calculated conventionally. (**f**) HMM sleep versus conventional sleep for each fish during day and night. r and p-value are for Pearson correlation for the fit to the line y=x.

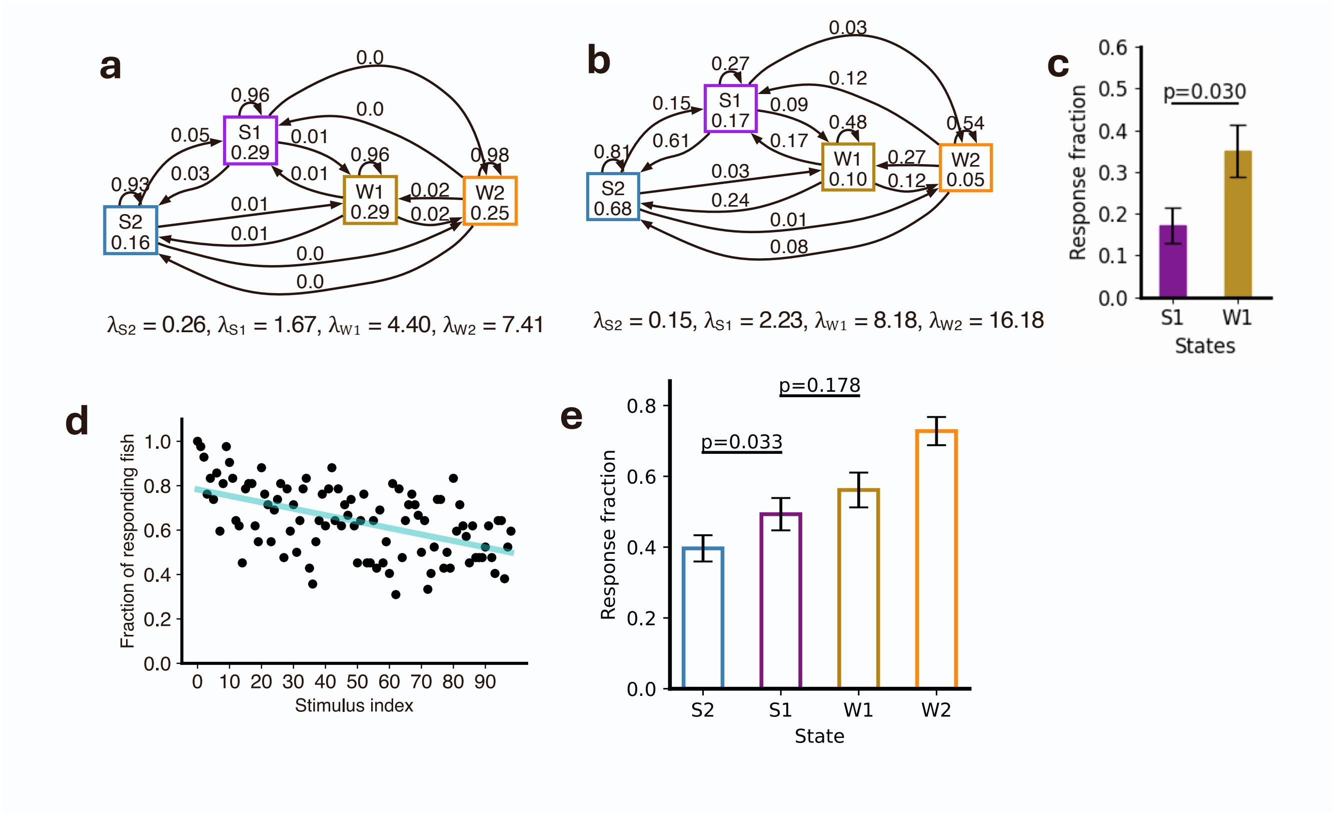

Figure S6: **HMM parameters for sleep deprivation and arousal experiments.** (**a**) Mean transition diagram and λ values for the HMM fit to sleep deprivation data. (**b-e**) Arousal threshold experiment. (**b**) Mean transition diagram and λ values for the HMM fit to arousal experiment data. (**c**) Response fractions from state S1 versus state W1, both corresponding to activity level of 5 s/min in the bin preceding the stimulus bin. (**d**) Fraction of fish responding to stimuli every five minutes throughout the tapping experiment. Cyan line shows the regression fit. (**e**) Separate model fits to individual fish: the response fraction for S1 was significantly higher than for S2. The p-values shown were calculated on response fractions from these states (p values for arcsin transformed response fractions from these states were 0.034 and 0.344, respectively).

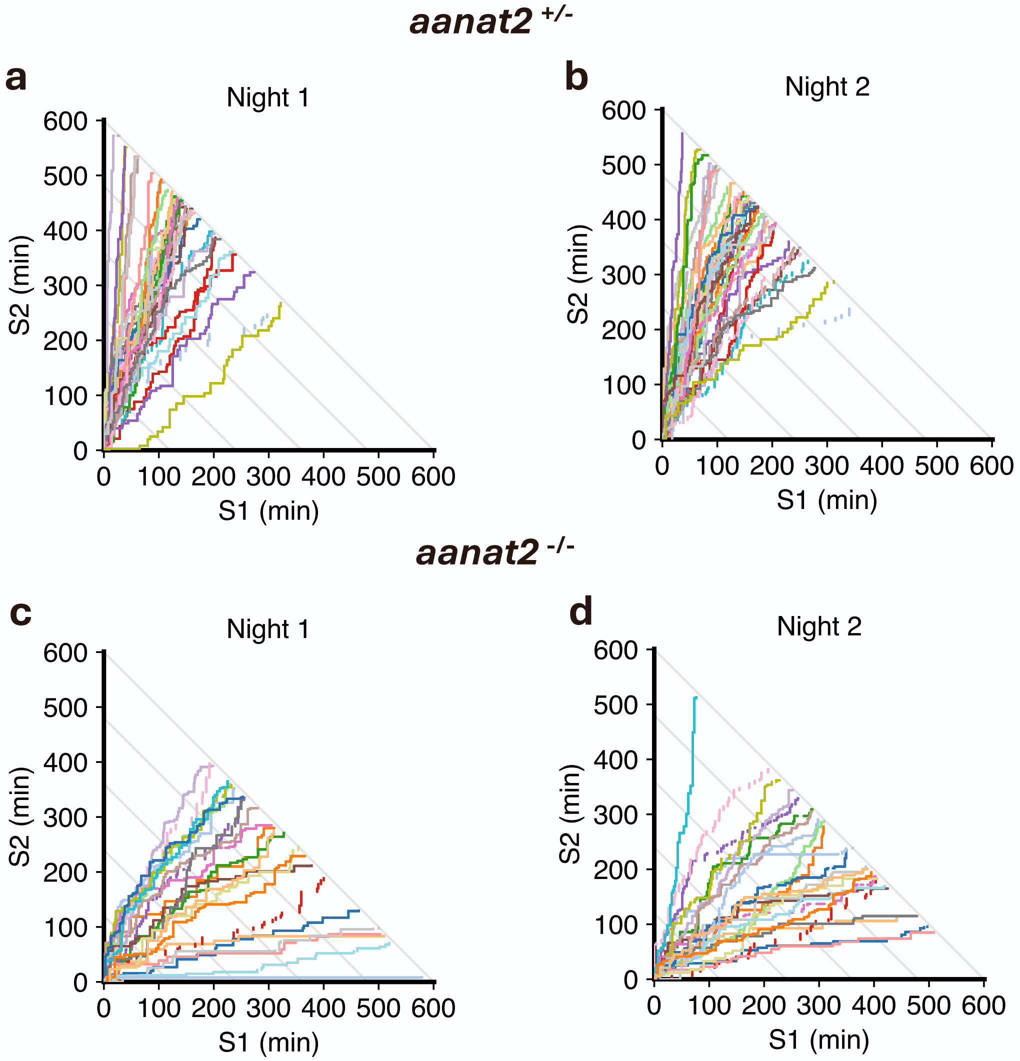

Figure S7: **Comparison of S1 and S2 state sequences during night for *aanat2*$^{+/-}$ and *aanat2*$^{-/-}$ fish.** (**a-b**) *aanat2*$^{+/-}$ fish. (**c-d**) *aanat2*$^{-/-}$ fish. Colors represent different fish. The increase in S1 relative to S2 in *aanat2*$^{-/-}$ fish is clearly apparent.
